# Optimization of CD34⁺ hematopoietic stem and progenitor cell isolation from human peripheral blood and evaluation of short-term culture conditions

**DOI:** 10.1016/j.mex.2026.104109

**Published:** 2026-08-20

**Authors:** Małgorzata Dąbrowska-Bovijn, Piotr Pobiarzyn, Aneta Skoniecka, Agnieszka Kowalska, Michał Pikuła, Aleksandra Rutkowska

**Affiliations:** a Division of Anatomy and Neurobiology, Medical University of Gdańsk, Gdańsk, Poland; b Division of Embryology, Medical University of Gdańsk, Gdańsk, Poland; c Department of Biochemistry, Gdańsk University of Physical Education and Sport, Gdańsk, Poland

**Keywords:** Immunomagnetic sorting, Density gradient centrifugation, Stem/progenitor cell enrichment

## Abstract

The recovery of viable, high-purity CD34⁺ cells from peripheral blood remains challenging for research applications. This study describes an optimisation process beginning with traditional Ficoll-based density gradient centrifugation and progressing to SepMate™ technology, combined with different magnetic separation systems and culture conditions, to establish an improved protocol for CD34⁺ cell isolation and subsequent culture for further research use. Early trials focused on cell concentration and seeding densities in stationary culture. Comparison of different density-gradient approaches indicated that the multiple centrifugation steps and prolonged processing times associated with Ficoll-based enrichment reduced CD34⁺ cell purity and viability compared with enrichment using SepMate™ tubes with Lymphoprep™. Column-based separation involving plunger-mediated elution was associated with reduced viability in the tested samples, although the effects of elution pressure could not be separated from processing duration or donor-specific variability. Despite multiple optimisation steps across enrichment, magnetic separation, and culture conditions, expansion of the cultured cells was not achieved. The methodological adjustments described here aim to improve the early handling of CD34⁺ cells from peripheral blood to provide a more reliable starting population for downstream culture studies and may facilitate the establishment of similar workflows in other laboratories.

• The use of SepMate™ tubes for PBMC isolation improved cell viability and recovery compared with Ficoll-based density gradient centrifugation.
• Different magnetic separation systems and culture conditions were evaluated to maintain cell viability and purity during culture.
• Agitation during culture supported maintenance of viable cells; however, after seven days the total cell number remained low and did not exceed the initial cell input.

## Graphical abstract

**Figure:**
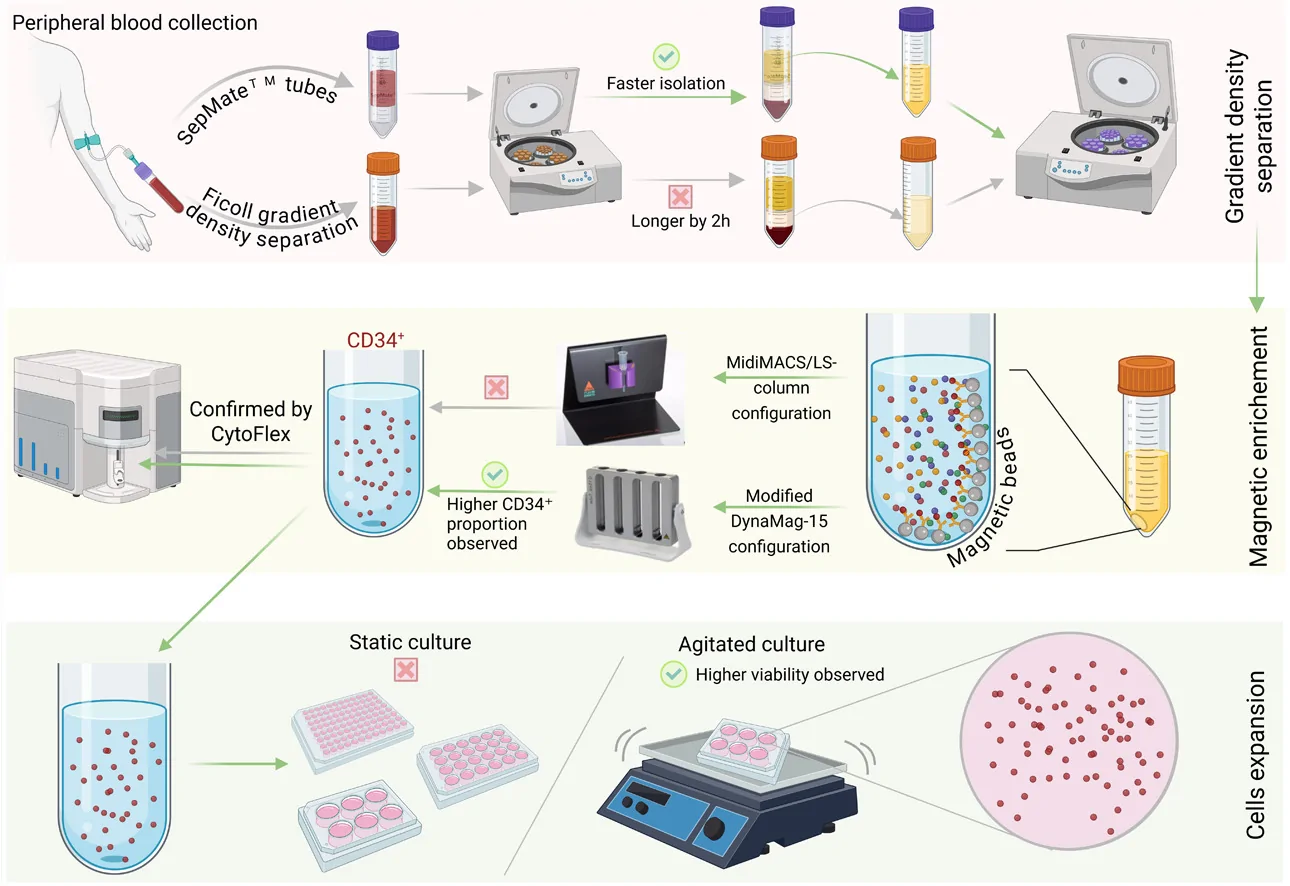

Specifications table.

**Table:** 

**Subject area**	Immunology and Microbiology			
**More specific subject area**	Hematopoietic stem and progenitor cell isolation			
**Name of your method**	Isolation of CD34⁺ cells from human peripheral blood			
**Name and reference of original method**				
**Resource availability**	**Product name**	**Company**	**Fluorochrome**	**Catalog number**
	CD34 MicroBead Kit UltraPure, human	Miltenyi BioTec	N/A	130-100-453
	MidiMACS™ Separator and Starting Kits	Miltenyi BioTec	N/A	130-042-302
	LS Columns	Miltenyi BioTec	N/A	130-042-401
	Ficoll	Merck	N/A	GE17-1440-02
	Phosphate buffer saline (PBS) 1X (without Calcium and Magnesium), 6 × 500 ml	Diag- Med	N/A	21-040-CV
	Ethylenediaminetetraacetic acid (EDTA)	Thermo Fisher Scientific (Invitrogen)	N/A	15575020
	SepMate™−50 (IVD)	Stemcell Technologies	N/A	85450
	Lymphoprep™	Stemcell Technologies	N/A	18061
	Invitrogen™ DynaMag™−15 Magnet	Thermo Fisher Scientific (Invitrogen)	N/A	12-301-D
	Mouse Anti-Human CD3 (clone SK7 (Leu-4)	BD Bioscience	APC—Cy™7	557832
	Mouse Anti-Human CD19 (clone SJ25C1)	BD Bioscience	BV605	562653
	Mouse Anti-Human CD34 (clone 581)	BD Bioscience	BV421	562577
	Mouse Anti-Human CD45 (clone HI30)	BD Bioscience	PerCP-Cy™5.5	564105
	Annexin V Apoptosis Detection Kit I	BD Bioscience	FITC	556547
	StemMACS™ HSC Expansion Media, human	Miltenyi BioTec	N/A	130-100-473
	StemMACS™ HSC Expansion Cocktail, human	Miltenyi BioTec	N/A	130-100-843

## Background

CD34 is commonly used to enrich heterogeneous populations of CD34⁺ hematopoietic stem and progenitor cells (HSPCs) from bone marrow, mobilized peripheral blood, umbilical cord blood and, less frequently, blood from donors who have not undergone mobilization. Conventional enrichment generally involves isolation of peripheral blood mononuclear cells (PBMCs), followed by magnetic selection of the CD34⁺ fraction using antibodies. Although these procedures are established, their sequential use requires repeated centrifugation, washing, transfer, labelling and elution, which may reduce cell recovery and affect the population available for culture.

These limitations are particularly relevant when small blood volumes are processed because circulating CD34⁺ cells are rare. Ficoll separation can support successful enrichment and culture of CD34⁺ cells; Merling et al., for example, isolated and cultured CD34⁺ cells from non-mobilized peripheral blood before reprogramming them into induced pluripotent stem cells [1]. Nevertheless, density gradient processing is not quantitatively neutral. In a rat bone-marrow comparison, Ficoll density-gradient centrifugation caused substantial mononuclear-cell loss and reduced recovery of colony-forming hematopoietic progenitors relative to alternative separation procedures [2]. Comparisons of conventional Ficoll separation, SepMate™ tubes and cell preparation tubes have shown differences in PBMC recovery, population composition and functional measurements, although immediate viability was similar [3]. In porcine bone-marrow preparations, the centrifugal force used during density-gradient isolation influenced viable-cell recovery, apoptosis, intracellular reactive oxygen species and colony-forming progenitor yield [4]. These studies do not establish direct Ficoll toxicity towards peripheral blood CD34⁺ cells, but indicate that prolonged processing, repeated centrifugation and multiple transfers may reduce yield or introduce variability.

Label-free microfluidic cell-enrichment technologies are also being developed. A closed, continuous-flow microfluidic device has been used to enrich lymphocytes from leukapheresis material [5]. Inertial microfluidics has enabled high-throughput separation of white blood cells from diluted whole blood [6], while deterministic lateral displacement has been used to enrich primary human skeletal progenitor cells based on their physical properties [7]. These studies demonstrate the potential of microfluidics for less manually intensive upstream cell processing. However, the cited systems target lymphocytes, total white blood cells or skeletal progenitor cells and have not been validated for direct selection of CD34⁺ HSPCs from non-mobilized peripheral blood. They should therefore be regarded as emerging upstream enrichment strategies rather than direct substitutes for CD34-targeted magnetic selection.

The present protocol was developed for small-scale, low-throughput basic research using limited volumes of non-mobilized peripheral blood and equipment commonly available in cell culture laboratories. It is not intended for therapeutic cell production under Good Manufacturing Practice conditions or presented as a clinically validated HSPC expansion procedure. The workflow compares conventional Ficoll isolation with separation using SepMate™ tubes and Lymphoprep™, evaluates centrifugation conditions for cell concentration, compares magnetic enrichment systems with and without columns, and assesses maintenance of the recovered cells in brief experimental culture.

The methodological contribution of this study is not a new CD34⁺-selection principle, but a sequential evaluation of established processing steps when applied to limited volumes of non-mobilized peripheral blood. Relative to a conventional workflow based on Ficoll-Paque isolation followed by column-based magnetic selection, the evaluated modifications included the use of SepMate™ tubes with Lymphoprep™ to shorten PBMC isolation, adjustment of post-isolation centrifugation conditions to reduce cell loss, comparison of an LS-column configuration with a modified column-free DynaMag-15 configuration using the same Miltenyi CD34 MicroBeads, and evaluation of static and agitated short-term culture. We hypothesized that reducing processing time and unnecessary handling steps would improve recovery and viability. The study therefore documents the performance and limitations of the tested configurations as practical guidance for laboratories working with small numbers of peripheral-blood CD34⁺ cells.

## Method details

### Collection of peripheral blood from donors

Peripheral blood was collected into EDTA-coated vials from healthy adult donors who had not undergone hematopoietic stem and progenitor cell mobilization.

### Density gradient separation of PBMCs

Initial experiments used conventional Ficoll-Paque density gradient separation. Peripheral blood was diluted with cold calcium- and magnesium-free PBS, pH 7.2, supplemented with 2 mM EDTA. In the documented experiments, 15 mL blood was combined with 35 mL buffer to obtain 50 mL diluted sample, whereas 27 mL blood was combined with 73 mL buffer to obtain 100 mL diluted sample. The diluted blood was divided between two or four 50 mL conical tubes, respectively, each containing 25 mL Ficoll-Paque. Approximately 25 mL diluted blood was carefully layered over the Ficoll in each tube. Samples were centrifuged at 400 × g for 40 min using slow acceleration and no brake.

Following centrifugation, the PBMC-containing interphase was transferred to fresh 50 mL conical tubes and brought to a final volume of 50 mL with cold PBS containing 2 mM EDTA. Cells were centrifuged at 300 × g for 10 min. For platelet depletion, the resulting pellet was resuspended in 50 mL buffer and centrifuged at 200 × g for 10 min. The pellet was subsequently resuspended in 25 mL buffer, passed through a 30 µm nylon mesh to remove aggregates, counted, and centrifuged again at 300 × g for 10 min before magnetic labelling.

In subsequent experiments, SepMate™−50 tubes with Lymphoprep™ were used to shorten the PBMC isolation procedure. Whole blood was diluted 1:1 with cold PBS supplemented with 2% fetal bovine serum (FBS). Fifteen millilitres of Lymphoprep™ (the density gradient medium) was added through the central hole in the insert of each SepMate tube, and no >27 mL of diluted blood was pipetted quickly down the side of each tube. The blood from a single donor was divided among several SepMate tubes. For example, 37 mL whole blood produced approximately 74 mL diluted sample and was distributed among three SepMate tubes, corresponding to approximately 24.7 mL diluted sample per tube. Samples from different donors were not pooled.

SepMate tubes were centrifuged at 1200 × g for 10 min, at room temperature, with the brake enabled. LymphoprepTM prevented mixing of the layers after centrifugation. Granulocytes and red blood cells pelleted at the bottom of the tube, whereas PBMCs remained above the insert. By one smooth motion PBMCs were harvested to fresh 50 mL conical tubes. Cells were then washed twice with 25 mL of cold PBS containing 2% FBS at 300 × g for 10 min 9room temperature). The use of SepMate tubes reduced the PBMC isolation stage from approximately 3 h to approximately 1 h by shortening centrifugation and eliminating manual aspiration of the interphase. The isolation conditions and corresponding cell recovery are summarized in Table 1.

**Table 1:** Comparison of cell recovery and CD34⁺ cell isolation efficiency using Ficoll density separation and different magnetic isolation methods, including SepMate tubes with Invitrogen and Miltenyi magnets. Trials were performed prior to the evaluation of CD34⁺ cell expansion. Each workflow was evaluated once using a single donor sample (n = 1 biological replicate per workflow).

	Magnetic isolation using Ficoll	Ultra-gentle magnetic isolation using Ficoll	Magnetic isolation using SepMate Tubes & Invitrogen magnet	Magnetic isolation using SepMate Tubes & Miltenyi magnet	Magnetic isolation of CD34^+^ for expansion culture
**Whole blood (ml)**	35	40	37	42	38
**Supernatant (number of cells)**	N/A	N/A	N/A	33.6 × 10^6^	6.48 *10^6^
**Before magnetic separation (number of cells)**		15 *10^6^	22.4 *10^6^	N/A	30.7 *10^6^
**After magnetic separation: CD34^+^ (Total viable cells recovered in the magnetically retained fraction (Trypan Blue count))**		7.2 *10^4^	N/A	12 *10^4^	88 *10^4^
**After magnetic separation: Flow through (number of cells)**		2.7 *10^6^	N/A	45.36 *10^6^	29 *10^6^

### Magnetic labelling and magnetic separation

Magnetic labelling was performed for both separation configurations using the CD34 MicroBead Kit UltraPure, human (Miltenyi Biotec, cat. no. 130-100-453). Cells were resuspended in 300 µL of PBS containing 2% FBS per 10⁸ total cells. FcR Blocking Reagent (100 µL) and CD34 MicroBeads UltraPure (100 µL) were added per 10⁸ total cells, and the suspension was incubated for 30 min at 4°C. Cells were subsequently washed with 10 mL of PBS containing 2% FBS by centrifugation at 350 × g for 10 min.

The labelled cells were processed using one of two magnetic-separation configurations. In the column-based configuration, cells were separated using an LS Column placed in a MidiMACS™ Separator (Miltenyi Biotec). Column-based separation using the MidiMACS™ separator and LS column was based on the high-gradient magnetic cell-separation principle described by Miltenyi et al. [8] Before sample application, the LS column was equilibrated with 3 mL of buffer. After loading the cell suspension, the column was washed three times, each time with 3 mL of buffer to remove unlabeled cells. Subsequently, the magnetically retained cells were eluted from the column using 5 mL of buffer after removal of the column from the magnetic field.

In the modified column-free configuration, cells labelled with the same Miltenyi CD34 MicroBeads were transferred to a 15-mL tube in a final volume of 5 mL and placed in a DynaMag™−15 magnet (Thermo Fisher Scientific, cat. no. 12301D). After incubation for 3 min at room temperature, the magnet and tube were inverted in one continuous motion to remove the unretained fraction. For a second separation, the tube was removed from the magnet, topped up with 5 mL of fresh buffer and incubated in the magnet for 1 min at room temperature. Subsequently, the supernatant was removed as described above. The retained cells were ready for further usage.

This represented a laboratory-developed combination of Miltenyi CD34 MicroBeads with the DynaMag-15 magnet rather than use of an Invitrogen CD34-selection kit or a manufacturer-validated separation protocol.

### Flow cytometry and gating strategy

The antibody panel comprised anti-human CD34–BV421 (clone 581), CD45–PerCP-Cy5.5 (clone HI30), CD3–APC—Cy7 (clone SK7) and CD19–BV605 (clone SJ25C1). Annexin V–FITC and propidium iodide were used to evaluate cell viability and apoptosis. Antibody catalogue numbers and suppliers are provided in the resource table.

Single-stained compensation controls were prepared for each fluorochrome in the panel. Compensation matrices were calculated in CytExpert software and subsequently verified manually.

Unstained cells were used to assess autofluorescence and define the negative populations for surface-marker gates. For Annexin V and propidium iodide analysis, quadrants were defined using unstained, Annexin V–FITC-only and propidium iodide-only controls. These gates were then applied consistently to classify viable (Annexin V⁻/PI⁻), early apoptotic (Annexin V⁺/PI⁻) and late apoptotic or dead (Annexin V⁺/PI⁺ or Annexin V⁻/PI⁺) events [9].

**Fig. 1:**
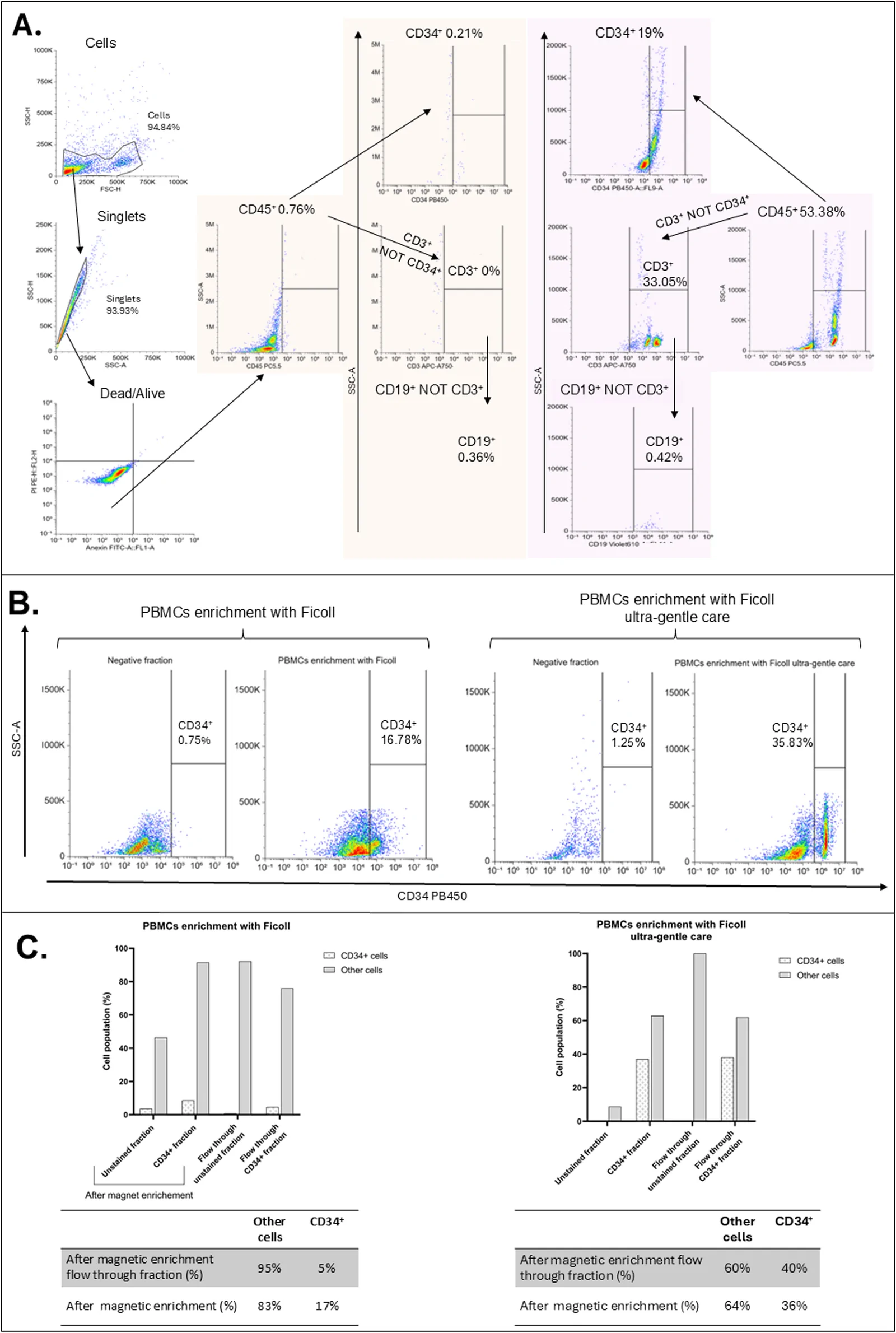
Enrichment and characterisation of CD34⁺ cells isolated from PBMCs using Ficoll. Representative plots from one donor are shown. CD34⁺ cells were labelled using CD34 MicroBead Kit UltraPure, (human) reagents and enriched by magnetic-activated cell sorting (MidiMACS™ Separator and Starting Kits, with LS columns), followed by analysis on a CytoFLEX flow cytometer. A) Gating strategy. Cell populations were first gated based on forward and side scatter parameters to exclude debris and doublets. Next, dead, late apoptotic, and early apoptotic cells were excluded using Annexin V/PI staining. Fluorescence gates were established using negative control samples for each surface marker to determine background autofluorescence levels (dot plots on orange background). Labelled samples were then analysed relative to the negative controls to identify positive cell populations. CD45⁺ cells were selected to define the hematopoietic parental population, from which CD34⁺, CD3⁺, and CD19⁺ subsets were determined (dot plots on pink background). B) Representative flow cytometry plots showing CD34⁺ expression in magnetically enriched cell fractions. Cells were analysed after magnetic separation on a CytoFLEX flow cytometer either without staining (negative control) or after staining with anti-CD34 antibody. The gated populations represent the proportion of CD34⁺ cells detected within the enriched fraction. A relatively low proportion of CD34⁺ cells was obtained after magnetic separation (∼17% of all cells). In comparison, using the ultra-gentle isolation procedure resulted in ∼36% CD34⁺ cells, demonstrating the higher efficiency of this optimised method. C) Bar graphs show the percentage distribution of CD34⁺ cells and other cell populations among 10,000 gated events acquired from the magnetically retained and flow-through fractions. Percentages were calculated from a fixed acquisition of 10,000 gated events. Samples labelled “unstained fraction after magnetic enrichment” represent cells collected after magnetic separation without antibody labelling, while “CD34^+^ fraction after magnetic enrichment” indicates cells labelled with anti-CD34 antibody to assess magnetic labelling. Bar graphs from the flow-through fraction show the efficiency of magnetic separation. The percentage of CD34⁺ cells for PBMCs enrichment with Ficoll was approximately ∼4,6% in the flow-through fraction, whereas after magnetic separation, it was ∼8,6%, indicating relatively low magnetic separation efficiency and perhaps not strong enough magnetic labelling. The approach of the ultra-gentle separation procedure resulted in an increased proportion of CD34⁺ cells in comparison to other cells. After magnetic separation, approximately ∼37% of CD34⁺ cells were found, which was a gain in the amount of cells in comparison to ∼8,6% of CD34+ cells found in ordinary Ficoll gradient density separation. However, the loss of CD34⁺ cells in the flow-through fraction was very high (∼62%). Each isolation condition was evaluated once using cells from a single donor (n = 1 biological replicate per condition). Values represent individual observations.

These antibodies were selected based on the gating strategy designed to identify hematopoietic stem and progenitor cells (CD34^+^) within peripheral blood mononuclear cells (PBMCs) (Fig. 1A). Following exclusion of debris and doublets based on forward and side-scatter parameters, live cells were identified using Annexin V/propidium iodide (PI) staining. CD45-positive cells were then selected to define the hematopoietic parental population. Within the CD45⁺ population, CD34⁺ cells were identified as a population enriched in hematopoietic stem and progenitor cells. CD34 expression alone does not distinguish primitive hematopoietic stem cells from more differentiated progenitor populations or establish functional stemness. To further characterise lymphocyte subsets within the remaining CD34^−^ fraction and assess the purity of magnetic labelling and cell isolation, sequential exclusion gating was applied. T cells were identified as CD3^+^ cells within the CD45^+^CD34^−^ population. Subsequently, B cells were defined as CD19^+^ cells within the CD45^+^CD34^−^CD3^−^ population, thereby excluding both CD34^+^ progenitor cells and CD3^+^ T cells [10]. Gates for positive populations were defined relative to unstained controls. Data acquisition and analysis were performed using a CytoFLEX flow cytometer and the corresponding analysis software. A graphical representation of the gating strategy is presented in Fig. 1A.

Data acquisition was carried out on a CytoFLEX flow cytometer (Beckman Coulter), and compensation matrices were calculated using CytExpert software. Spectral overlap between fluorochromes was corrected automatically and subsequently verified manually. Unless otherwise specified, flow-cytometric results are reported as percentages of gated events from a fixed acquisition of 10,000 events per sample. Where raw event numbers are shown, they represent acquired flow-cytometric events and not absolute cell counts or values extrapolated from the analysed sample volume. Absolute total viable cell numbers were determined separately by manual haemocytometer counting with Trypan Blue exclusion.

### Post-isolation culture conditions

Cells were cultured for 7 days in StemMACS™ HSC Expansion Medium XF supplemented at 1:100 with StemMACS™ HSC Expansion Cocktail, human (Miltenyi Biotec, cat. no. 130-100-843), containing recombinant human stem cell factor, Flt3 ligand and thrombopoietin. No cytokines other than those contained in the StemMACS cocktail were added. Cultures were maintained in uncoated, non-tissue-culture-treated plates at 37°C in a humidified atmosphere containing 5% CO₂.

For the preliminary static density screen, CD34⁺-enriched cells were seeded in 96-well U-bottom plates at 800, 2000, 4000 or 8000 cells in a final volume of 100 µL per well. Cells were also evaluated in 24-well flat-bottom plates at 1000, 3500, 7000 or 14,000 cells in a final volume of 200 µL per well and in six-well plates at 2500 cells (5000/mL) in a final volume of 500 µL per well.

For the direct static-versus-agitated comparison presented in Fig. 4, cells obtained from the same donor-derived cell suspension were seeded in six-well plates at density of 1 × 105 cells per well in 2 mL of supplemented medium per well. Static cultures remained undisturbed, whereas agitated cultures were placed horizontally on a Grant Instruments’ PMS-1000i Microplate Shaker with an orbital diameter of 2 mm. The shaker was positioned inside the incubator, the plate was placed on a shaker platform, and agitation was applied continuously at 150 rpm for 7 days.

Half-volume medium replacement was performed on days 2, 4 and 6. At each feeding, prior to careful removal of 1 mL of medium from the surface of the culture, cells were first allowed to settle for approximately 10 min. Subsequently, an equal volume of fresh StemMACS medium containing the expansion cocktail was added. No intermediate samples were removed for analysis. On day 7, the entire culture was recovered by pipetting the whole volume of each well into 15 ml tubes. Then wells were rinsed with fresh media twice (500 ml each time) and added into the 15 ml tubes. Counting using Trypan Blue was performed prior to centrifugation (5 min, 350 g). After removal of the supernatant from tubes, samples were ready for the analysis by flow cytometry using the panel described above.

### Statistics

A biological replicate was defined as an independent donor sample. Unless otherwise stated, each optimisation comparison and the proof-of-concept culture evaluation were performed once using cells from a single donor (n = 1), without donor-level biological replication. The experiments were performed sequentially as exploratory methodological optimization steps and were not designed as replicated comparative experiments. Accordingly, the results are therefore presented as raw descriptive observations. Means, standard deviations, error bars and inferential statistical tests were not calculated because biological replication was not available. Differences between conditions are therefore described without implying statistical significance or generalizability beyond the conditions tested. Because different donor samples were used for sequential optimization steps, observed differences may reflect both procedural and donor-related variability.

## Method validation

### Yield, purity and viability differences between peripheral blood isolation using Ficoll vs SepMate tubes

In two initial Ficoll-based experiments, 15 and 27 mL of peripheral blood yielded 21 × 10⁶ and 34 × 10⁶ PBMCs, respectively. Following magnetic separation, approximately 40,000 and 70,000 total viable cells were recovered in the magnetically retained fractions, as determined by manual haemocytometer counting with Trypan Blue exclusion. In the original manuscript, these total post-separation cell counts were incorrectly treated as CD34⁺ cell counts when calculating the values of 0.19% and 0.20%. These percentages did not represent either the physiological frequency or the absolute recovery of CD34⁺ cells and have therefore been removed. The proportion of CD34⁺ cells within the retained fractions was assessed separately by flow cytometry and was approximately 16% (Fig. 1B). >20% of the CD34⁺ population was apoptotic or dead immediately after magnetic labelling and separation (Fig. 3A).

The low proportion of CD34^+^ cells after magnetic separation following ordinary enrichment with Ficoll, combined with the high proportion of non-CD34⁺ cells, was concerning, particularly given the similar distribution observed in the flow-through fraction (∼95% non-CD34⁺ vs. ∼5% CD34⁺; Fig. 1C). This finding suggested suboptimal handling and/or inefficient magnetic labeling and sorting, resulting in insufficient recovery of stem cells, with a comparable fraction lost in the flow-through. However, the flow-through fraction did not exhibit similarly elevated levels of dead cells (Fig. 3A), suggesting that CD34^+^cells may have remained trapped within the magnetic column, or the plunger-mediated elution may have been too aggressive, leading to reduced cell viability.

To address this issue, an ultra-gentle elution approach was implemented in subsequent isolation. This modification increased the proportion of recovered CD34⁺ cells compared with the standard procedure (∼36% vs. ∼17%, respectively; Fig. 1C). This improvement reflected enhanced purity of the eluted fraction rather than increased overall cell recovery. Indeed, the ultra-gentle approach resulted in a greater loss of CD34⁺ cells in the flow-through fraction (∼40% vs. ∼5% with the standard procedure; Fig. 1C), indicating that the gain in purity was achieved at the expense of cell yield. In addition, overall cell viability remained poor, with approximately 60% of the CD34⁺ population undergoing late apoptosis following magnetic separation (Fig. 3C). Annexin V/PI staining showed that the number of late apoptotic cells exceeded 5000, whereas fewer than 5000 viable cells were recovered (Fig. 3C). Although the ultra-gentle approach improved the purity of the isolated CD34⁺ population, the reduced cell yield and poor post-isolation viability indicated that an alternative isolation strategy was required.

The ultra-gentle elution procedure did not improve cell viability and was associated with a higher proportion of late apoptotic cells in the tested sample. This observation does not support the initial assumption that reducing plunger pressure alone would improve viability. Because each configuration was evaluated once and differed in both handling and processing duration, the relative contributions of elution pressure, processing time and donor-specific variability cannot be distinguished. The poor recovery and viability observed with this configuration prompted subsequent evaluation of the shorter SepMate™-based PBMC isolation workflow.

**Fig. 2:**
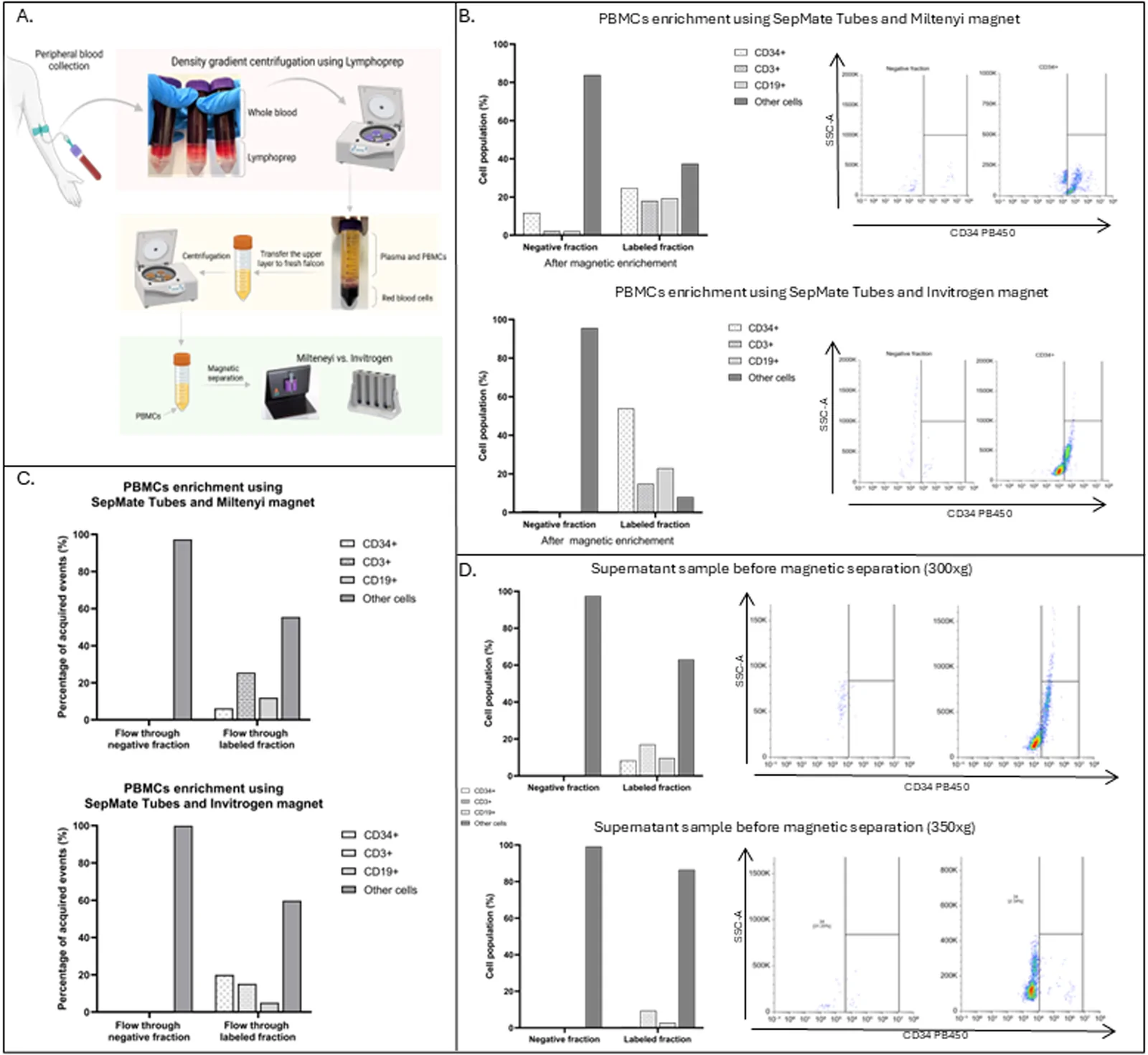
Workflow and efficiency of CD34⁺ cell enrichment from peripheral blood using SepMate tubes. A) Schematic overview of the experimental workflow. Peripheral blood was collected from donors and PBMCs were isolated by density gradient centrifugation using SepMate™ tubes with Lymphoprep™. This method allows centrifugation for 10 min (1200x*g*), enabling rapid recovery of the PBMC-containing layer. Subsequently, CD34⁺ cells were enriched using magnetic separation and compared between two systems: the Miltenyi Biotec system (LS column with plunger-based elution) and the Invitrogen™ modified column-free DynaMag-15 configuration using Miltenyi CD34 MicroBeads. The three SepMate™−50 tubes shown represent aliquots of a single donor sample after 1:1 dilution with PBS containing 2% FBS; samples from different donors were not pooled B) Purity of CD34⁺ cell enrichment following magnetic separation using the two systems. Bar graphs show the distribution of unstained cells and cells stained with the antibody panel (anti-CD45, anti-CD34, anti-CD19, and anti-CD3). Cells negative for all antibodies were classified as “other cells”. Representative flow cytometry plots illustrate the proportion of CD34⁺ cells obtained after magnetic enrichment. The same analysis was performed for samples enriched using both magnetic systems. The MidiMACS/LS-column configuration yielded 23,42% CD34^+^cells, while the modified DynaMag-15 configuration yielded 52,64% CD34^+^ cells. C) Cell distribution in the flow-through fraction. Analysis of cell populations detected in the flow-through fraction following magnetic separation suggested the distribution of cells that were not retained during magnetic enrichment or were not successfully magnetically labelled. Percentages were calculated from a fixed acquisition of 10,000 gated events. In the Miltenyi system, the loss of CD34^+^ cells in the flow-through fraction was low, corresponding to approximately 6.2%,while with modified DynaMag-15 configuration CD34^+^ loss reached ∼20%. D) Cell loss in the discarded supernatant prior to magnetic separation. Analysis of cell populations present in the supernatant fraction, which was discarded before the magnetic separation, revealed that ∼8% of CD34^+^cells were lost during centrifugation at 300x*g*. Increasing the centrifugation speed to 350x*g* reduced the proportion of CD34+ cells detected in the supernatant to ∼2%. Each enrichment workflow was evaluated once using cells from a single donor (n = 1 biological replicate per condition). Values represent individual observations.

CD34⁺ cells isolated following either PBMC isolation protocol were subsequently enriched using the Miltenyi Biotec magnetic separation system. Regardless of the downstream magnetic enrichment strategy, PBMC isolation using SepMate™ tubes outperformed the conventional Ficoll protocol by reducing processing time and improving the quality of the starting cell population. Compared with the standard Ficoll method (∼5% CD34⁺ cells), SepMate™ isolation increased the purity of the final CD34⁺ fraction to ∼25% when combined with the MidiMACS/LS-column configuration and to ∼58% when combined with the modified DynaMag-15 configuration (Fig. 2B).

**Fig. 3:**
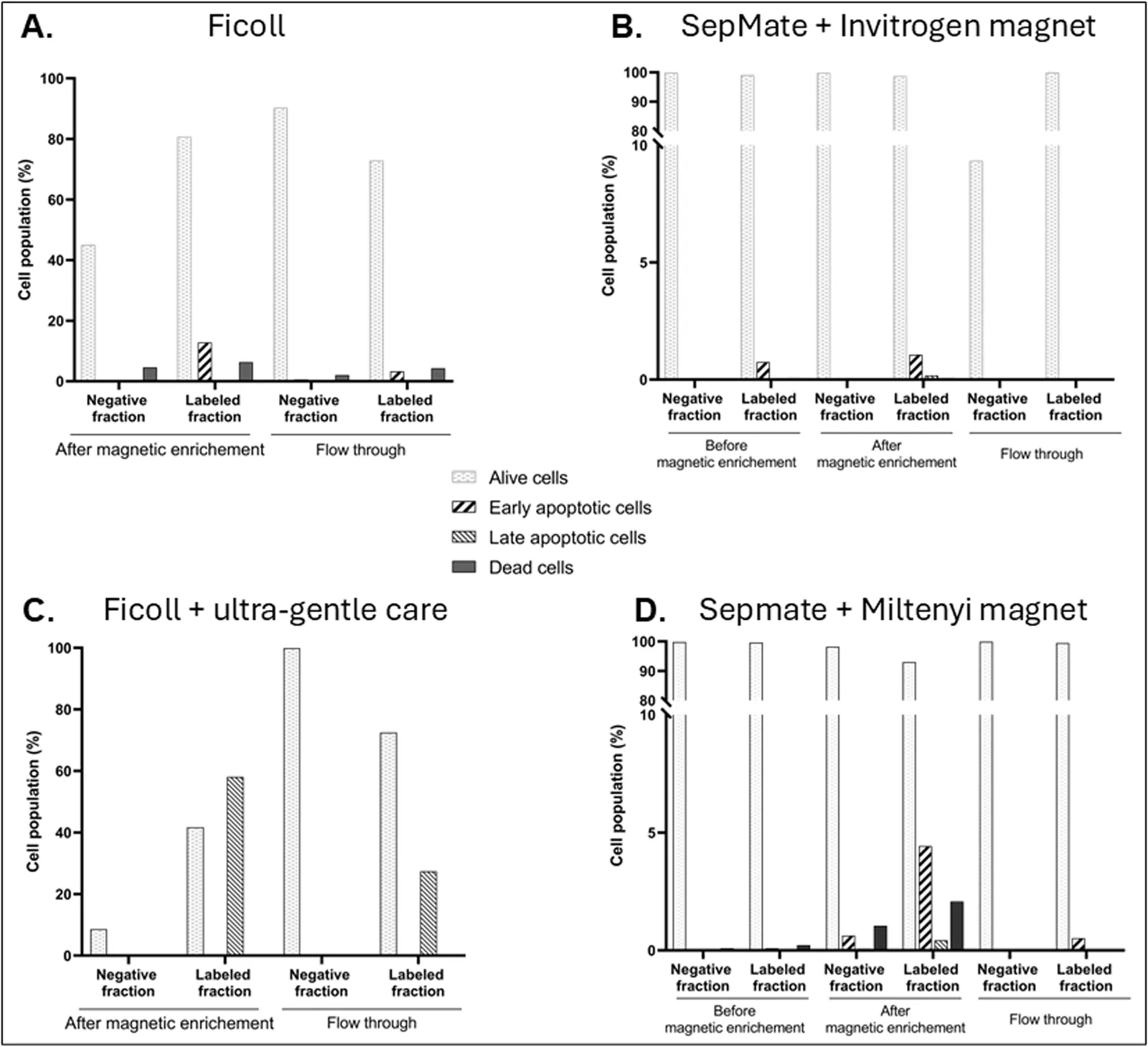
Annexin V analysis of PBMC viability following different enrichment and magnetic separation strategies. Annexin V staining was used to evaluate cell viability in different fractions, including cells recovered after magnetic separation and flow-through fractions, in both unstained samples and CD34⁺-stained samples. Bars represent the number of gated events classified as alive, early apoptotic, late apoptotic, or dead cells. A) PBMCs enrichment using Ficoll density gradient centrifugation followed by magnetic separation. While the overall number of gated events of viable cells was high, fractions collected after magnetic separation using the MidiMACS/LS-column configuration contained a higher proportion of early apoptotic and dead cells compared with other fractions. B) Assessment of cell viability during PBMCs processing using SepMate™ tubes combined with the Invitrogen magnetic separation system. A reduced proportion (by ∼90%) of early apoptotic cells was observed in the fraction collected after magnetic separation compared with samples processed using Ficoll-based density gradient enrichment. C) PBMCs enrichment using a modified “ultra-gentle” Ficoll protocol followed by magnetic separation. Annexin V staining was used to assess the effect of this modified handling procedure on cell viability in post-magnet and flow-through fractions. Despite gentler handling during buffy coat collection and plunger elution, the fraction of cells recovered after magnetic separation contained a higher proportion of late apoptotic cells than viable cells. D) Viability analysis of PBMCs processed using SepMate™ tubes combined with the Miltenyi magnetic separation system. The same PBMC enrichment method (SepMate™ tubes) was used to allow comparison between the wo magnetic-separation configurations. As viability was very high (above 98%) while using the Invotrogen system, the MidiMACS/LS-column configuration compromised the viability of the cells (most likely because of the usage of columns and the plunger). Additional comparison between SepMate™- and Ficoll-based PBMC isolation was performed using the Miltenyi magnetic system. Differences in the proportions of early apoptotic, late apoptotic, and dead cells were observed between the processing conditions. Usage of SepMate™ Tubes decreased the early apoptotic cells percentage by ∼59%, late apoptotic cells by ∼96% and dead cells by ∼76%. Each processing condition was evaluated once using cells from a single donor (n = 1 biological replicate per condition). No inferential statistical comparisons were performed.

Although the ultra-gentle Ficoll protocol generated a higher proportion of CD34⁺ cells (∼36%) than the SepMate™–Miltenyi workflow (∼25%), this advantage was offset by substantially poorer cell viability (Fig. 3). In contrast, SepMate™-based isolation improved cell viability while also reducing the proportion of CD34⁺ cells lost in the flow-through fraction by approximately 50% compared with the ultra-gentle Ficoll protocol (Fig. 2C vs. Fig. 1C). Together, these findings suggested that shortening the PBMC isolation procedure using SepMate™ tubes provides an improved starting platform for subsequent CD34⁺ cell enrichment, irrespective of the magnetic separation system employed.

### Comparative analysis of magnetic separation systems

The transition to SepMate™ tubes and the elimination of Ficoll improved initial cell viability. A critical comparison was then conducted between the Invitrogen™ DynaMag™−15 and the Miltenyi Biotec magnetic systems. In the single samples analysed, CD34⁺ cells represented 23.42% of gated events after column-based separation and 52.64% after separation using the DynaMag™−15 configuration (Fig. 2B). The column-based sample contained a higher proportion of apoptotic and dead cells than the DynaMag™−15 sample (Fig. 3D). CD34⁺ purity and cell viability were therefore considered as separate outcomes. Because each configuration was evaluated once, these observations do not establish the superiority of either separation approach.

Flow cytometry analysis confirmed that while the MidiMACS/LS-column configuration yielded fewer CD34^+^ cells in the flow-through fraction (Fig. 2C). In this exploratory comparison, the modified DynaMag-15 configuration was associated with a higher proportion of CD34⁺ cells and greater post-separation viability than the MidiMACS/LS-column configuration.

### Cell concentration and centrifugation efficiency

Proper cell concentration was associated with better CD34⁺ cell recovery. Quantitative analysis of the supernatant after plasma centrifugation revealed a substantial loss of approximately 50,000 CD34^+^ cells in total supernatant volume when standard centrifugation speeds (300x*g*) were used (Fig. 2D). Flow cytometry dot plots (Side Scatter Pulse Height (SSC—H) vs. CD34 PB450-A) of the supernatant confirmed the presence of a distinct CD34^+^ population that failed to pellet. Increasing the centrifugation force from 300x*g* to 350x*g* in subsequent trials successfully minimised this loss, reaching approximately 12,000 CD34^+^cells in a total supernatant volume (Fig. 2D). This improvement of density gradient centrifugation ensured a larger starting population for downstream expansion.

### Cell recovery and viability after post-isolation culture

**Fig. 4:**
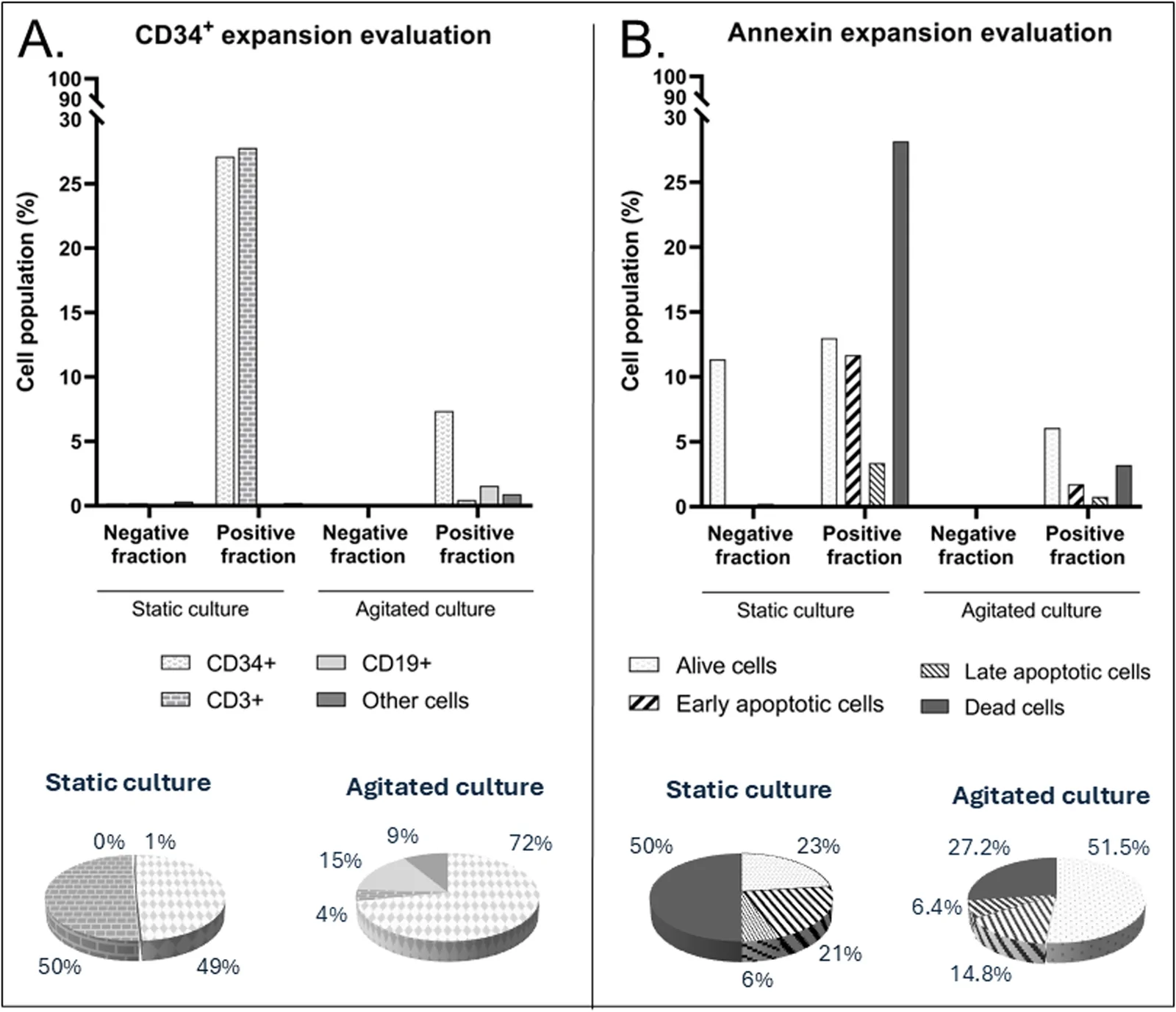
Cell composition and viability following seven days of static or agitated culture. A) The proportion of CD34⁺ cells was lower in cultures maintained with agitation while compared to cultures grown under static conditions (∼7.36% vs. ∼27.12%, respectively). Populations of CD3⁺ cells were also reduced in the agitated culture compared to the static culture (∼0.44% vs. ∼27.78% respectively), while amount of CD19⁺ cells was slightly increased in the agitated culture (∼1.56% in agitated culture, while 0.06% in static culture). Pie charts depict the relative distribution of cell populations within each sample. Percentages were calculated relative to the total analyzed cell population (100%), corresponding to the bar graph above. This presentation illustrates the overall cellular composition following static and agitated culture conditions. B) Viability of the same cell populations shown in panel A was assessed using Annexin V staining. The percentage of dead cells exceeded 28.16% in the static culture, whereas approximately 3.2% dead cells were detected in the agitated culture. Additionally, cells cultured under agitation exhibited a higher overall viability percentage compared to the static culture (∼51% vs. ∼23%, respectively), as presented on the pie charts below graphs. Pie charts depict the relative distribution of cell viability categories within each sample. Percentages were calculated relative to the total analyzed cell population (100%) corresponding to the bar graph above. Static and agitated cultures were evaluated using cells obtained from a single donor (n = 1 biological replicate). Values represent individual observations. the two conditions were generated by splitting the same donor sample.

In early trials, despite testing a broad range of seeding densities, no increase in cell number was observed after 7 days. At the end of the evaluation of the expansion period, flow cytometry was used to monitor changes in cell populations and viability. In this single-donor comparison, culture under agitation at 150 rpm was associated with a higher proportion of viable cells than static culture (Fig. 4B). Nevertheless, the final cell number remained below the initial input under both conditions, indicating that neither condition supported net expansion. Because only one agitation speed was evaluated in a single biological experiment, these observations do not establish whether 150 rpm was optimal or detrimental to CD34⁺ cell expansion. However, the absolute cell yield remained below the target level in both tested cultures (static vs. agitated) (Fig. 4A).

Pie charts derived from flow cytometry data showed that at day 7, the agitated culture maintained a higher percentage of viable cells, compared with the stationary culture, which was dominated by dead and late apoptotic populations (Fig. 4B). Despite the improvement in viability, the agitation of 150 rpm setting was identified as too aggressive for optimal CD34^+^ cell expansion, as it induced substantial cell death and prevented achievement of the desired expansion yield, while other lymphatic cells managed better in this conditions (Fig. 4A). Differences in the obtained cell populations and viability levels are presented in Table 2.

**Table 2:** Cell recovery, composition and viability following seven days of culture. Cells were cultured under static vs. agitated conditions. Cell viability, apoptotic status, and expression of CD34, CD19, and CD3 were compared between both conditions. Marker-positive populations were quantified after gating on viable cells. Total cell numbers were determined by Trypan Blue exclusion immediately after culture. Following Annexin V/PI staining and flow cytometric acquisition, the total number of recovered events (including viable, early apoptotic, late apoptotic, and dead cells) was compared with the initial cell count. Static and agitated culture conditions were evaluated using cells from a single donor (n = 1 biological replicate). The two conditions were generated by splitting the same donor sample. CD34⁺ percentage was calculated as the number of CD34⁺ gated events divided by the total number of recovered gated events after labelling × 100.

	Before labelling	After labelling								
	Total amount of cells	Total amount of cells	CD34^+^	CD34^+^(percentage)	CD19^+^	CD3^+^	Alive cells (total)	Early apoptotic cells (total)	Late apoptotic cells (total)	Dead cells (total)
**Static culture, day 7**	15 *10^4^	2761	1356	49.1%	3	1389	23.1%	20.8%	6%	50.1%
**Agitated culture, day 7**	2380	513	368	71.718%	78	22	51.6%	14.8%	6.4%	27.2%

### Limitations

The first part of the study aimed to establish an optimized isolation workflow. The second part provides a proof-of-concept evaluation of the optimized protocol under expansion conditions. Although, this study has several limitations that should be considered when interpreting the results. First, the overall yield of CD34⁺ cells obtained from peripheral blood was relatively low. This is expected because CD34⁺ hematopoietic stem and progenitor cells circulate at very low frequencies in the peripheral blood of healthy non-mobilized adults [11] Consequently, the limited starting population restricts both downstream analyses and the potential for successful expansion [12]. Consequently, the limited starting population restricts both downstream analyses and the potential for successful expansion. Second, no expansion of CD34⁺ cells was observed under the culture conditions tested. The cultures were supplemented with the manufacturer-recommended StemMACS™ HSC Expansion Cocktail containing FLT3 ligand, SCF and TPO. Therefore, absence of cytokine support does not explain the lack of expansion. Successful culture of isolated human CD34⁺ cells has been reported using other culture systems. For example, Bonde et al. [13] described CD34⁺ cell isolation followed by culture, screening of medium components and establishment of long-term cultures. Consequently, our negative result should not be interpreted as indicating that CD34⁺ cells cannot be expanded following immunomagnetic isolation. Potential contributors include the low viable CD34⁺ cell input, incomplete enrichment, donor-specific variability and cumulative cell loss or stress during isolation and culture handling. Because these factors were not examined independently and the culture experiment was performed using cells from a single donor, the cause of the lack of expansion cannot be established from the present study. Future studies should evaluate alternative cytokine combinations or concentrations, different starting cell densities and culture formats across multiple donors, with absolute viable CD34⁺ cell counts and colony-forming assays. Additional optimization of culture parameters may therefore be required. Several important variables known to influence stem cells' survival and expansion were not explored in this study, including alternative cytokine combinations or concentrations beyond the manufacturer-supplied cocktail, oxygen tension (e.g., hypoxic culture conditions), potential cytotoxic effects associated with the isolation procedures, and the application of dynamic culture systems such as bioreactors.

All optimisation comparisons and culture evaluations were based on a single donor per experiment (n = 1), without biological replication. The observed differences may therefore reflect donor-specific variation and do not establish the superiority of one method or support broadly generalisable recommendations. The findings should instead be interpreted as exploratory troubleshooting observations that identify workflow configurations requiring validation in independent donors.

Studies in other experimental settings show that culture interventions can affect CD34 expression and/or Annexin V/PI-defined apoptosis. l-carnitine altered CD34 expression and apoptosis in MACS-enriched rat bone-marrow CD34+ cells, whereas co-culture with bone-marrow-derived mesenchymal stromal cells altered apoptosis in K562 leukemia cells [14,15]. These models differ substantially from normal human non-mobilized peripheral-blood CD34+ HSPCs and therefore provide only indirect context for the present findings.

Another limitation is that the comparison of enrichment strategies was restricted to two magnetic separation systems. Although the results provide useful insights, additional CD34⁺ enrichment technologies could be evaluated in future studies to determine whether higher recovery or purity can be achieved. The column-free arm used Miltenyi CD34 MicroBeads with a DynaMag-15 magnet, representing a non-standard cross-platform configuration. Consequently, its observed performance should not be interpreted as validation of an established Invitrogen CD34-isolation workflow. The lack of biological replication precludes estimation of variability and formal comparison between workflows. In addition, where different donor samples were used for sequential optimization steps, procedural effects cannot be separated from donor-related variability. Furthermore, donor-to-donor variability may influence the efficiency of cell isolation, viability, and expansion potential, which could affect the reproducibility of the results. However, the objective of each optimization step was to identify the most promising protocol to carry forward to the subsequent stage, rather than to statistically compare all candidate methods.

Finally, the analysis relied primarily on flow cytometry markers and viability assays (Annexin V/PI). While CD34^+^ expression is widely used for the identification of hematopoietic stem and progenitor cells, CD34^+^ alone does not fully confirm functional stem cell potential. A more comprehensive immunophenotyping panel, including markers such as CD7 (one of the earliest T/NK lineage markers), CD10 (early lymphoid progenitors) and CD38 (developmental stage marker), would allow better discrimination between primitive hematopoietic stem cells and more differentiated progenitor populations.

## Ethics statements

The Bioethics Committee for Scientific Research at Medical University of Gdansk (Poland) approved the human participant study under the licence number: NKBBN/457/2019 and KB/233-93/2026.

## CRediT author statement

**MDB:** Methodology, Investigation, Data curation, Formal analysis, Visualization, Writing – original draft; **PP:** Methodology, Investigation, Writing – review & editing; **AS:** Investigation, Writing – review & editing; **AK:** Investigation, Writing – review & editing; **MK:** Resources, Writing – review & editing; **AR:** Conceptualization, Methodology, Resources, Supervision, Project administration, Funding acquisition, Writing – review & editing.

## Declaration of interests

The authors declare that they have no known competing financial interests or personal relationships that could have appeared to influence the work reported in this paper.

## Data Availability

Data will be made available on request.
